# Supplementary material for: Reporting quality and spin in abstracts of randomized clinical trials of periodontal therapy and cardiovascular disease outcomes
Source: PLoS One. 2020 Apr 17;15(4):e0230843. doi: 10.1371/journal.pone.0230843 (PMC7164582; doi:10.1371/journal.pone.0230843)
Supplement: S1 Table — (DOCX) [file pone.0230843.s001.docx]

S1 Table. List of the trial registries used in the search process

| Australian New Zealand Clinical Trials Registry (ANZCTR) |
| --- |
| Brazilian Clinical Trials Registry (ReBec) |
| Chinese Clinical Trial Register (ChiCTR) |
| Clinical Research Information Service (CRiS), Republic of Korea |
| ClinicalTrials.gov |
| Clinical Trials Registry – India (CTRI) |
| EU Clinical Trials Register (EU-CTR) |
| German Clinical Trials Register (DRKS) |
| Iranian Registry of Clinical Trials (IRCT) |
| ISRCTN.org |
| Japan Primary Registries Network (JPRN) |
| Sri Lanka Clinical Trials Registry (SLCTR) |
| The Netherlands National Trial Register (NTR) |
| Cuban Public Registry of Clinical Trials (RPCEC) |
| Thai Clinical Trials Registry (TCTR) |
| Pan African Clinical Trial Registry (PACTR) |
| Peruvian Clinical Trial Registry (REPEC) |
