# Supplementary figures and images for: Reporting quality and spin in abstracts of randomized clinical trials of periodontal therapy and cardiovascular disease outcomes
Source: PLoS One. 2020 Apr 17;15(4):e0230843. doi: 10.1371/journal.pone.0230843 (PMC7164582; doi:10.1371/journal.pone.0230843)

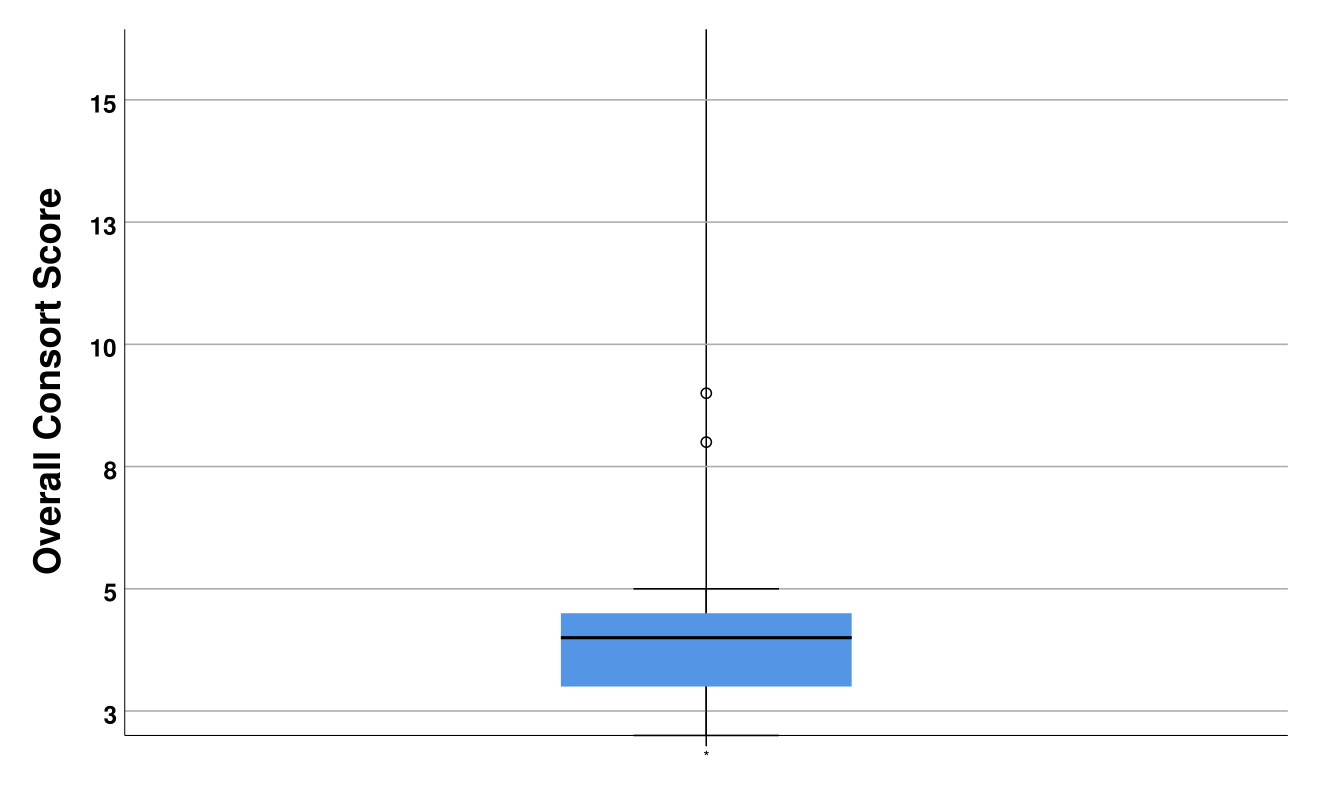

Supplement: S1 Fig — Articles were de-identified. Y-axis represents the fulfilled CONSORT items per article. (TIF) [file pone.0230843.s002.tif]

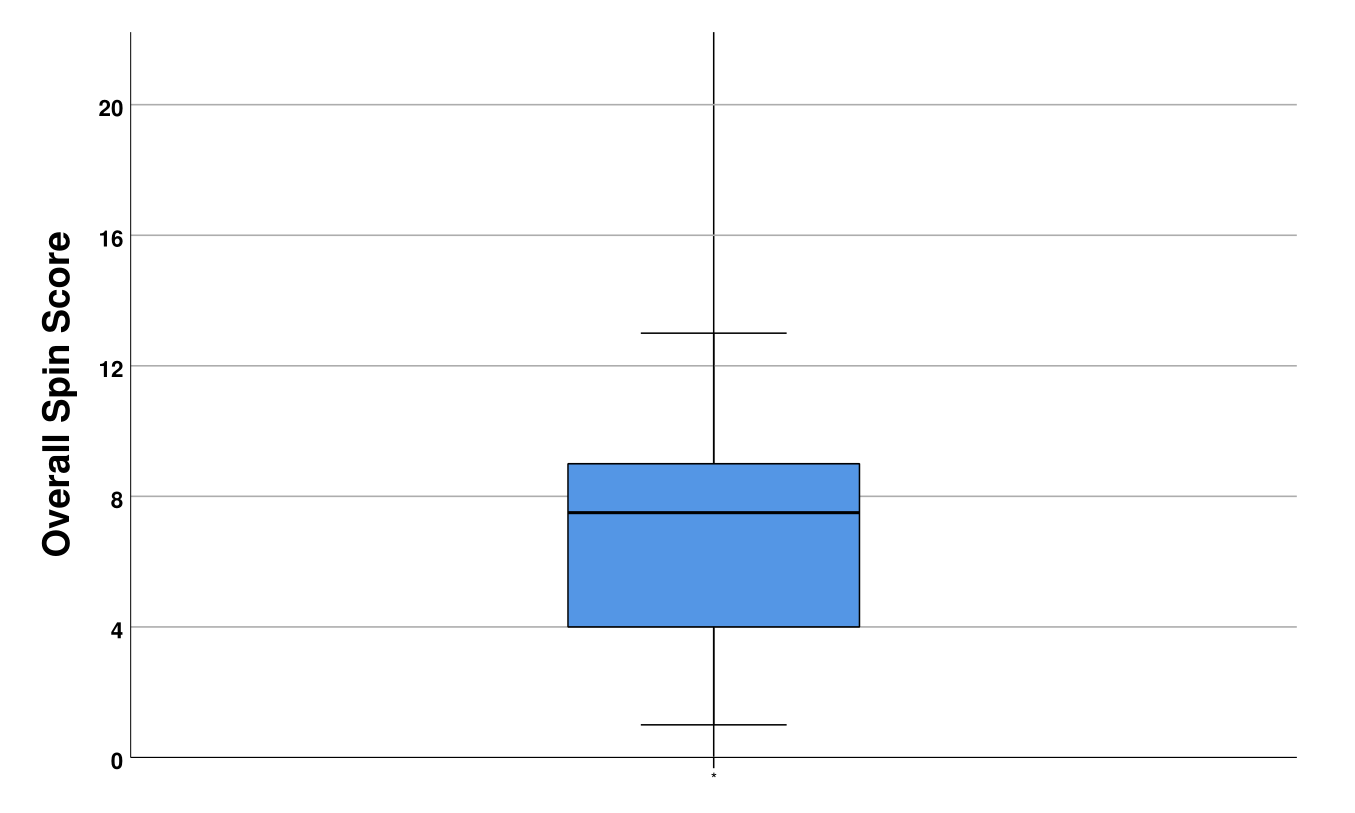

Supplement: S2 Fig — The articles were de-identified. Y-axis represents the numbers of fulfilled Spin checklist items. (TIF) [file pone.0230843.s003.tif]
